# Essential immune functions of fibroblasts in innate host defense

**DOI:** 10.3389/fimmu.2022.1058862

**Published:** 2022-12-15

**Authors:** Kellen J. Cavagnero, Richard L. Gallo

**Affiliations:** Department of Dermatology, University of California, San Diego, La Jolla, CA, United States

**Keywords:** fibroblast, mesenchymal, stromal, innate, immunity, barrier, defense, inflammation

## Abstract

The term fibroblast has been used generally to describe spindle-shaped stromal cells of mesenchymal origin that produce extracellular matrix, establish tissue structure, and form scar. Current evidence has found that cells with this morphology are highly heterogeneous with some fibroblastic cells actively participating in both innate and adaptive immune defense. Detailed analysis of barrier tissues such as skin, gut, and lung now show that some fibroblasts directly sense pathogens and other danger signals to elicit host defense functions including antimicrobial activity, leukocyte recruitment, and production of cytokines and lipid mediators relevant to inflammation and immunosuppression. This review will synthesize current literature focused on the innate immune functions performed by fibroblasts at barrier tissues to highlight the previously unappreciated importance of these cells in immunity.

## Introduction

**Figure 1:**
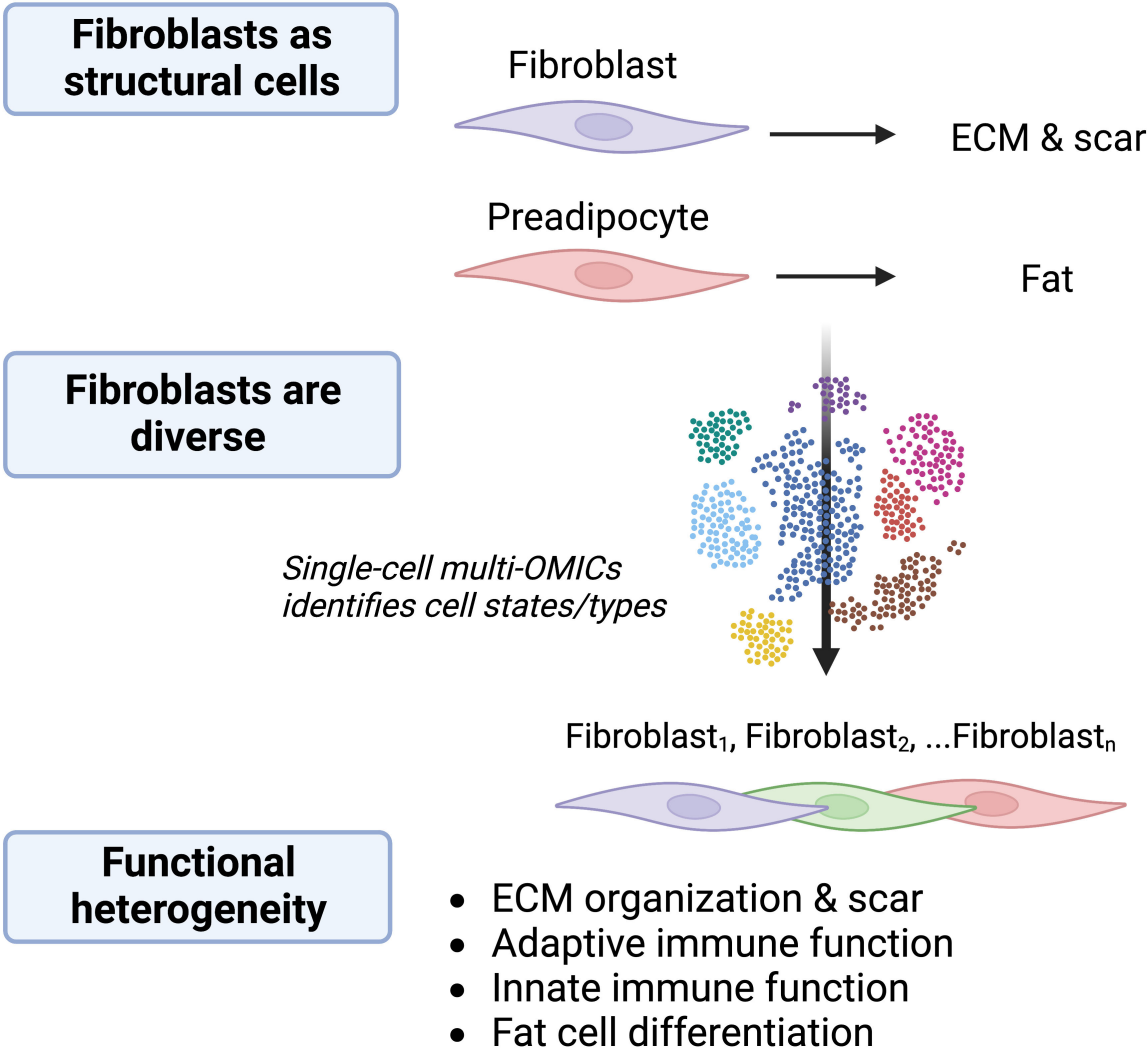
Fibroblasts are diverse and multifunctional. The understanding of cells with fibroblastic morphology is evolving. Fibroblasts were previously considered to be primarily structural cells responsible for production of ECM, acting in wound healing and fibrosis, and serving as stem cells for differentiation into structures such as fat. Single-cell multi-OMICS technology including scRNAseq has identified several fibroblast subtypes and activation states within and between tissues and disease states and revealed numerous potential distinct functions for these cells. Determining the functional significance of fibroblasts is an ongoing area of research. The choice of colors for fibroblasts illustrates that there are different functional subtypes/activation states.

The idea of functionally distinct fibroblasts subpopulations was suggested in the literature over 40 years ago (1). The heterogeneity of fibroblasts has since been confirmed through multiple approaches including genetic lineage-tracing and next-generation sequencing. Focusing first on their classical role in wound repair and development, discrete lineages of fibroblasts with unique functions were identified in the skin (2, 3). Fibroblast phenotypic diversity across murine tissues was later revealed using bulk transcriptomic and epigenetic sequencing approaches (4). More recently, single-cell RNA sequencing (scRNAseq) has enabled unbiased investigation into intra- and inter-tissue fibroblast heterogeneity. For example, scRNAseq analysis of fibroblasts from naïve and *Cutibacterium acnes* infected skin revealed a mixture of eight distinct fibroblast subtypes and states, four of which were unique to infected skin (5). Three of these populations expressed low levels of genes related to classical fibroblast matrix organization function while others expressed high levels of genes related to immunity. Furthermore, integration of scRNAseq datasets from several inflamed tissues in mice and humans showed that while tissue-specific characteristics can be defined for some fibroblastic cells, all tissues are composed of three broad categories of fibroblasts: progenitor fibroblasts, steady-state fibroblasts, and inflammation-associated fibroblasts (6). These observations suggest that some subsets of fibroblasts perform immune functions that are somewhat specific to the tissue and type of perturbation. Overall, current information from analysis of gene expression by fibroblasts suggest they can exist in several functional states with a range of activities in organism development, scar formation, and immunity (**Figure 1**).

In the following review, we will discuss the emerging and somewhat unexpected revelations that some fibroblasts are direct participants in innate host defense and inflammation. To illustrate this, we will synthesize *in vitro* work done over the last several decades that has demonstrated that barrier tissue fibroblasts act in pattern recognition, respond to cytokines, and elicit innate immune effector functions. The latter half of this review will then focus on work that has begun to unveil the functional significance of fibroblast immune activity during infections of skin, gut, and lung, as well as in inflammatory diseases and cancer. The capacity of fibroblasts to educate lymphoid cells will not be discussed in detail as these indirect activities of fibroblasts in adaptive immunity were the subject of another excellent recent review (7).

## Fibroblasts sense danger

**Figure 2:**
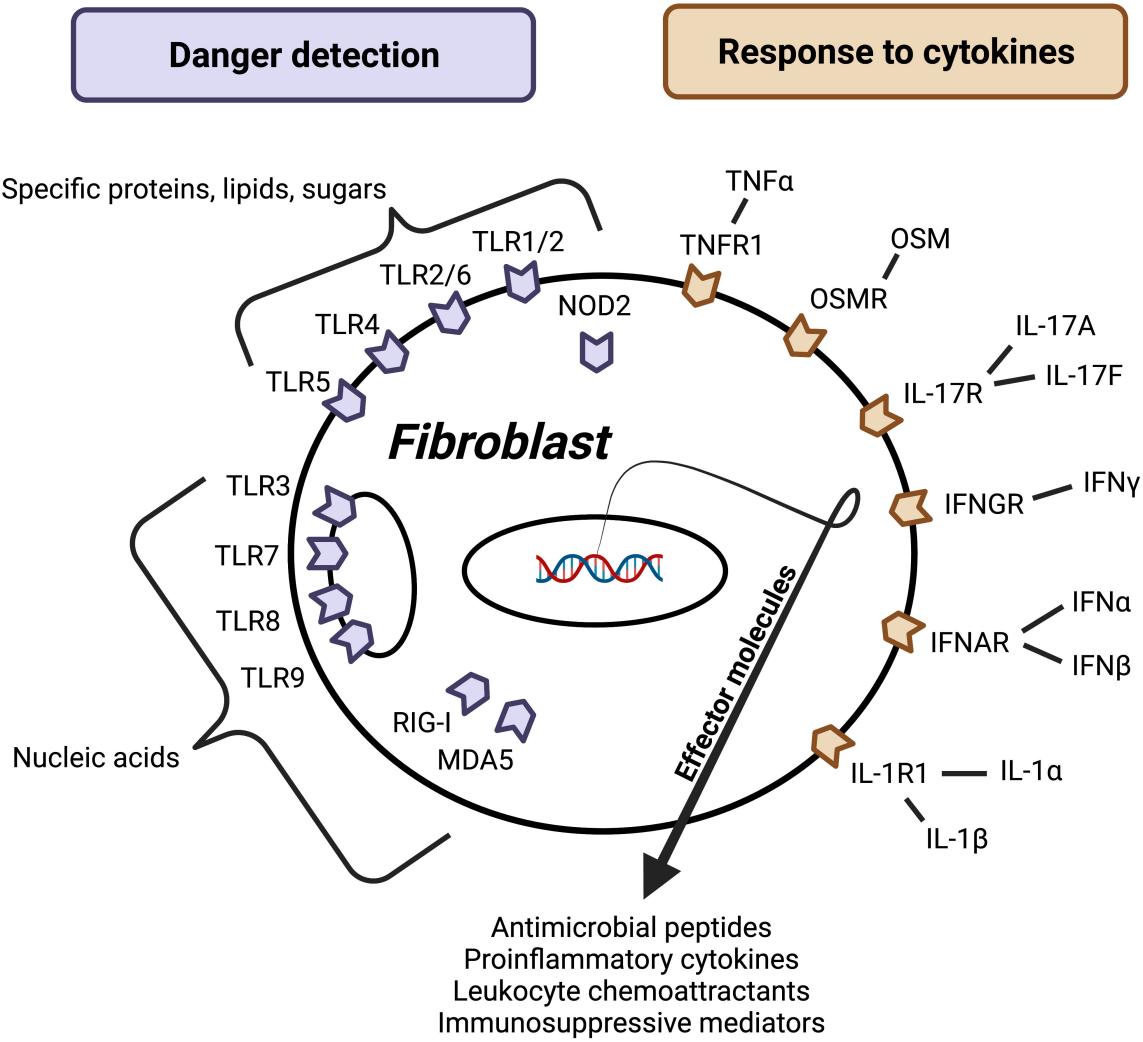
Factors regulating fibroblast effector function. Barrier tissue fibroblasts sense danger and respond to cytokines, leading to activation and release of effector molecules. Danger signals may be microbe- or host-derived and detected using surface, cytosolic, or endosomal receptors (purple). While these receptors can broadly be divided into a group that recognizes nucleic acids and group that recognizes certain molecules composed of proteins, lipids, and/or sugars, each receptor has evolved to recognize a unique set of ligands. Host-derived soluble protein signals called cytokines activate surface receptors (orange). Importantly, this figure represents a synthesis of barrier tissue fibroblast activating signals; more research is needed to understand how fibroblast activation varies between different fibroblast populations. Additionally, more work is needed to identify negative regulators and understand how other signals like neuronal factors, lipid mediators, and hormones impact barrier tissue fibroblast effector function.

A key feature of innate immunity is the ability to rapidly detect pathogen/microbial/danger-associated molecular patterns (PAMPs, MAMPs, and DAMPs) using pattern recognition receptors (PRRs) that initialize host defense against bacterial, viral, and fungal pathogens (8). This function has been generally attributed to front-line epithelial cells and tissue-resident classical immunocytes such as macrophages and dendritic cells. Numerous studies have now demonstrated that fibroblasts in the skin, gut, and lung express functional PRRs including membrane and endosomal toll-like receptors (TLRs) and cytoplasmic retinoic acid-inducible gene I (RIG-I)-like receptors and NOD-like receptors (NLRs) (**Figure 2**) (9–12).

One outcome of fibroblast pattern recognition is the secretion of inflammation mediators, and this response appears to depend on the molecule being detected, the tissue of origin, and the host of origin. Danger detection systems must be able to elicit a diversity of responses in order to promote defense against a broad array of pathogens. Stimulation of skin fibroblasts with ligands to TLR1/2, TLR2/6, TLR3, TLR4, TLR5, TLR7, TLR8, and TLR9 has been shown to promote secretion of the proinflammatory cytokine IL-6 and neutrophil chemokine CXCL8 (13, 14), while the TLR3 ligand Poly(I:C), but not ligands to other TLRs, was shown to induce expression of the leukocyte chemokines CXCL9 and CXCL10 by fibroblasts in the skin (15). Commensal and pathogenic microbes are typically unique to the epithelial organ and therefore the danger detections system must be specific to the location. Contrary to skin fibroblasts, ligands for TLR1/2, TLR2/6, and TLR4—but not TLR3, TLR5, TLR7, and TLR9—led to IL-6 secretion by intestinal fibroblasts (10, 16). Microbiota composition is also unique to the host, necessitating somewhat individualized pattern recognition capabilities. It has been observed that skin fibroblasts from different donors display a large range of responses to the TLR4 ligand lipopolysaccharide (LPS) independent of age and sex (4-7x difference between low and high responders) (17, 18).

Fibroblast PRR signaling is also important for cell-intrinsic antiviral immunity. Human skin fibroblast expression of TLR3 is necessary for mounting cell-autonomous antiviral responses to Zika virus infection (19). Intriguingly, the mechanisms underlying cell-intrinsic antiviral immunity in fibroblasts appear to differ from those employed by classical immunocytes. One study showed that skin fibroblasts from an individual with an autosomal recessive TLR3 deficiency demonstrated an impaired response to herpes simplex virus (HSV)-1 infection, while the individual’s peripheral blood mononuclear cells responded normally (20).

Taken together, these reports demonstrate that barrier tissue fibroblasts are poised to detect a variety of danger signals and initiate defense responses tailored to the environment and threat encountered. That fibroblasts in the skin, gut, and lung subepithelial stroma have functional pattern recognition activity further illuminates the multilayered defense system of barrier tissues and fills an important gap in knowledge that is currently not typically considered when modeling inflammatory pathways in these organs.

## Fibroblasts respond to cytokines

Effective innate immune cells must also be able to detect inflammatory mediators sent from other immune cells and propagate immune responses. Like conventional innate immunocytes, fibroblasts in the skin, gut, and lung have been shown to respond to a wide variety of proinflammatory cytokines known to be important for host defense (**Figure 2**).

IFNγ is produced largely by T helper 1 (Th1) cells and group 1 innate lymphoid cells (ILC1s) for defense against intracellular bacteria, viruses, and parasites (21). In 1984, Pfefferkorn demonstrated that *in vitro* stimulation of skin fibroblasts with IFNγ induces cell-intrinsic defense against the intracellular pathogen *Toxoplasma gondii* (22). IFNγ has also been shown to elicit skin fibroblast expression of the CXCR3 ligands CXCL9 and CXCL10 that are important for Th1 recruitment and maturation (14, 15). Moreover, a recent study found that a given fibroblast’s response to IFNγ is dependent upon its anatomic location. Specifically, murine skin fibroblast expression of CXCR3 ligands following IFNγ stimulation is decreased in cells isolated from paw skin compared to those from dorsal or ventral skin (23). Intriguingly, IFNγ and TLR ligands synergistically promote skin fibroblast expression of CXCL8 and CXCL10 (14), suggesting that fibroblasts are sentinel cells that integrate danger signals from immune cells and microbes.

TNFα is a proinflammatory cytokine that is critical for host defense and is clinically relevant to several barrier tissue chronic inflammatory diseases including psoriasis and inflammatory bowel disease (IBD) (24). Biologic TNFα inhibitors effectively treat patients with these conditions, though treatment increases risk of certain infections. Despite the clinical success of TNFα inhibitors, the precise mechanisms by which these therapeutics regulate immunity are unclear, but they may act in part by interfering in the capacity of fibroblasts to propagate inflammatory signals. In support of this, TNFα was initially found to induce IL-6 and CXCL8 in skin fibroblasts (25). Currently, a long list of inflammatory mediators are known to be expressed by TNFα-activated fibroblasts from the skin, gut, and lung and these include a vast array of proinflammatory cytokines and chemokines relevant to innate immunity (14, 26–28). Interestingly, one study found that Poly(I:C) primes lung fibroblasts to produce more IL-6 following a later TNFα exposure (29), suggesting that fibroblasts could potentially drive post-viral inflammatory conditions.

IL-17A is a proinflammatory cytokine produced by subsets of lymphocytes including T helper 17 (Th17) cells, γδT cells, and group 3 innate lymphoid cells (ILC3s), and it is important for defense against extracellular pathogens (30, 31). As with TNFα, IL-17A is associated with barrier tissue chronic inflammatory diseases including psoriasis and IBD, and drugs inhibiting IL-17A signaling are currently used to treat patients. While IL-17A is widely appreciated as a potent, albeit indirect, driver of neutrophil recruitment, the exact mechanisms underlying IL-17A-mediated immune responses are unclear but could potentially be in part through fibroblasts. In 1996, Fossiez and colleagues first cloned and purified recombinant human IL-17A and found that it induces IL-6, CXCL8, granulocyte colony stimulating factor (G-CSF), granulocyte-macrophage colony stimulating factor (GM-CSF), and prostaglandin E2 (PGE2) production in skin fibroblasts (32). Since this early discovery, skin, gut, and lung fibroblasts stimulated with IL-17A have been shown to produce additional inflammatory mediators including IL-1α and IL-1β (26, 28, 33–36). IL-17A is well-known for synergizing with proinflammatory cytokines by stabilizing mRNA and promoting translation (30). In barrier tissue fibroblasts, IL-17A has been found to synergize with TNFα, IL-1β, and Poly(I:C) (26, 33, 36–38). Intriguingly, conditioned medium from IL-17A- and TNFα-stimulated skin fibroblasts promotes γδT cell production of IL-17A, suggesting that fibroblasts contribute to the propagation of immune responses through bi-directional communication with IL-17A-producing lymphocytes (26).

IL-1R1 ligands, IL-1α and IL-1β, are proinflammatory cytokines secreted primarily by epithelial cells and macrophages, respectively. Like IL-17A and TNFα, IL-1α and IL-1β are associated with barrier tissue chronic inflammatory diseases and targeted in treatment, and inhibition of these mediators also increases risk of infection. Pioneering work in the 1980s revealed that IL-1, alone and in synergy with TNFα, induces lung fibroblast production of inflammatory mediators (28, 39–42). Others have since shown that IL-1β activates skin and gut fibroblasts (43, 44) and that IL-1β together with Poly(I:C) synergistically induces IL-6 and CXCL8 expression in lung fibroblasts (11). Elegant *in vitro* experiments have demonstrated the importance of IL-1-induced fibroblast activation in physiologically relevant settings. Lysates from damaged intestinal and lung epithelial cells drive IL-1α-dependent intestinal and lung fibroblast inflammatory mediator production, respectively, and conditioned media from IBD patient tissue explants elicits IL-1-dependent intestinal fibroblast secretion of neutrophil chemoattractants including CXCL1 and CXCL8 (11, 45, 46). Importantly, the effect of IL-1α on skin fibroblasts is lineage-specific. Skin fibroblasts arise from at least two distinct lineages (2). One lineage (*Lrig1* ^+^ and *Blimp1* ^+^) gives rise to the upper dermis (proximal to the epithelium) and includes papillary fibroblasts. The other lineage (*Dlk1* ^+^) gives rise to the lower dermis (distal to the epithelium) and includes reticular fibroblasts and preadipocytes. IL-1α-stimulated papillary fibroblasts produce more GM-CSF than IL-1α-stimulated reticular fibroblasts (47), indicating that cells derived from the upper dermal lineage are primed to interact with local keratinocyte epithelial cells.

Oncostatin M (OSM), a member of the IL-6 family of cytokines, has been linked to inflammatory barrier tissue diseases including psoriasis, atopic dermatitis, IBD, and allergic airway disease but has no defined role in host defense (48). Nevertheless, OSM has also been shown to promote skin, gut, and lung fibroblast immune activation and synergize with TNFα (49–51).

Collectively, these studies demonstrate that barrier tissue fibroblasts are equipped with the machinery necessary to propagate innate immune responses initiated by other tissue-resident cells for defense against several classes of pathogens.

## The immune effector functions of fibroblasts

**Figure 3:**
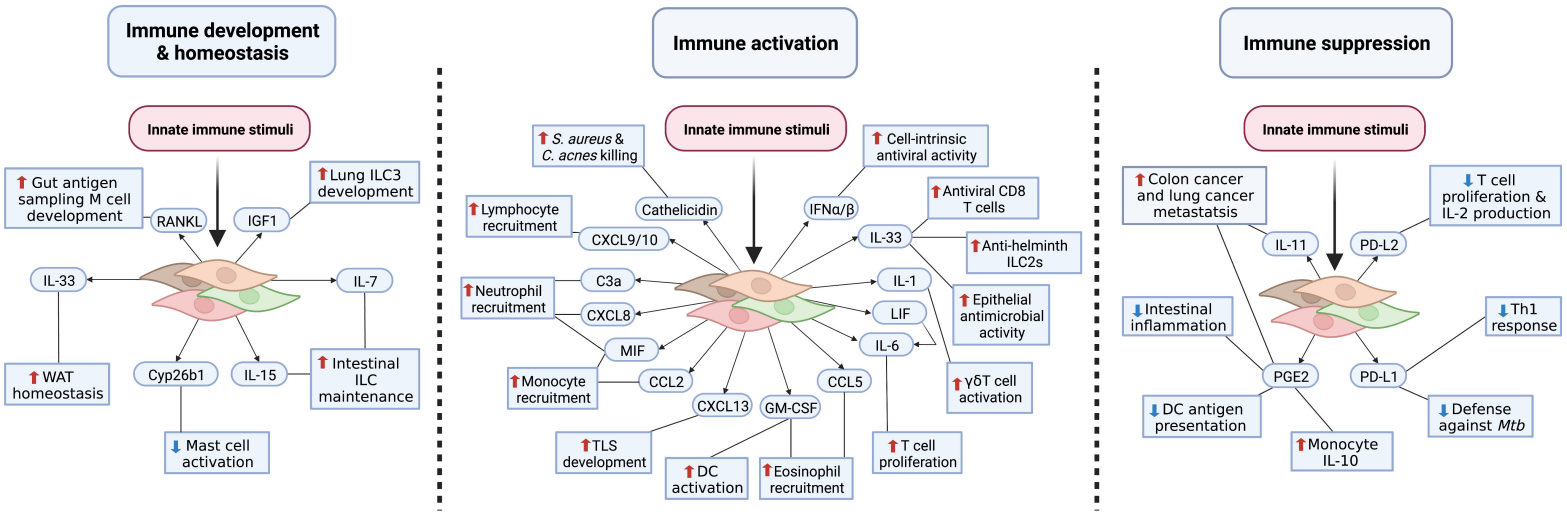
Fibroblast innate immune effector functions. Barrier tissue immune acting fibroblasts elicit innate immune functions required for development and homeostasis, immune activation, and immunosuppression. During steady-state conditions, fibroblasts produce cytokines and growth factors necessary for immunological priming and homeostasis (left panel). To potentially eliminate pathogens, fibroblasts release antimicrobial peptides, chemokines, and proinflammatory cytokines (middle panel). Left unchecked, this defense response could contribute to chronic inflammatory conditions. To limit excessive and protracted immune responses and prevent inflammatory disease pathogenesis, fibroblasts upregulate immune checkpoint molecules, suppressive bioactive lipid mediators, and anti-inflammatory cytokines; however, inappropriate fibroblast immunosuppression may increase risk of infection and cancer (right panel). This figure represents an amalgamation of proposed barrier tissue fibroblast innate immune effector functions. It is unclear whether each fibroblast subtype/state across tissues is capable of achieving an activation state that will enable these functions.

That some fibroblasts can sense danger and respond to a variety of inflammatory mediators has gained new relevance as there now is abundant evidence that fibroblasts can elicit antimicrobial activity, promote leukocyte recruitment, and secrete cytokines and bioactive lipids. These functions have relevance to organism immune system development and homeostasis, inflammation, and immunosuppression (**Figure 3**), thus demonstrating how fibroblasts are a key element of innate immune defense. Fibroblasts may also influence immunity through antigen presentation and matrix regulation; however, discussion of these mechanisms is beyond the scope of this review and have been reviewed elsewhere (52, 53). In this section, we will review the emerging innate immune effector functions of barrier tissue fibroblasts.

### Antimicrobial activity

Arguably the most critical aspect of host defense is the elimination of microbial threats through production of endogenous antibiotics. While direct antimicrobial activity is largely attributed to epithelial cells, neutrophils, and macrophages, there is a growing body of evidence demonstrating that fibroblasts are a key source of antimicrobials. The essential role of the cathelicidin antimicrobial peptide in the skin was demonstrated in 2001 (54). Subsequent *in vitro* studies suggested that microbes may induce fibroblast expression of cathelicidin as well as defensin antimicrobial peptides (55–58). In 2015, dermal fibroblasts belonging to a preadipocyte lineage (*Dlk1* ^+^) were shown to secrete large amounts of cathelicidin while undergoing adipogenesis induced by *Staphylococcus aureus* infection (59). Importantly, the antimicrobial function of these cells was necessary for the skin to resist infection. Production of cathelicidin by preadipocytes has more recently been shown to be potentiated by TLR2 and TLR4 ligands and retinoic acid (5, 60, 61). Further, the profibrotic cytokine TGFβ has been found to inhibit the host defense function of this fibroblast subset, and this mechanism may contribute to increased infections observed during ageing and obesity (states of elevated TGFβ production) (62, 63).

Subsets of fibroblasts have also been shown to display phagocytic capacity, hinting that some fibroblasts also possess intracellular killing mechanisms (64–66). Indeed, a recent investigation found that Sca-1^+^ lung fibroblasts produce reactive oxygen species and antimicrobial proteins and potently inhibit the growth of intracellular *Klebsiella pneumoniae* (66). Notably, several chemokines known to be produced by fibroblasts demonstrate direct antimicrobial activity *in vitro—*particularly CXCL9 and CXCL10*—*and therefore may be relevant to the ability of fibroblasts to kill bacteria (reviewed by (67)). In sum, these reports reveal that some fibroblasts are potent antimicrobial cells.

### Leukocyte recruitment and proinflammatory cytokine production

Another key aspect of host defense is the propagation of immune responses through chemokine and proinflammatory cytokine production. In response to innate immune stimulation, barrier tissue fibroblasts have been shown to secrete several proinflammatory cytokines and chemokines.

In 1996, Noso and colleagues published one of the earliest studies identifying barrier tissue fibroblasts as important regulators of immune cell recruitment (68). In this study, skin fibroblasts stimulated with TNFα were shown to promote eosinophil chemotaxis through GM-CSF and CCL5. A more recent study demonstrated that *Ng2* ^+^ skin pericytes (an endothelial-adjacent type of fibroblast) colocalize with neutrophils and monocytes following subcutaneous injection of TNFα, and *in vitro* transwell migration assays with umbilical pericytes indicated that these cells promote macrophage migration inhibitory factor (MIF)- and CXCL8-dependent neutrophil migration and MIF- and CCL2-dependent monocyte migration (69). Other studies showed that lung fibroblasts promote complement C3a-dependent neutrophil migration in an *in vitro* transwell migration assay (70), and that skin fibroblasts activated by IFNγ promote CXCL9- and CXCL10-dependent lymphocyte migration (23). Beyond leukocyte recruitment, chemokine production by fibroblasts is known to be important for the development of lymphoid structures (reviewed by (71)). Of note, innate immune stimuli—including IL-1, TNFα, IL-17A, and type 1 IFN—induce barrier tissue fibroblast production of CXCL13 required for lymphoid tissue inducer cell clustering and germinal center formation (72–78).

Some fibroblasts from barrier tissues have been found to express the IL-1 family members IL-33, IL-1α, and IL-1β. IL-33 and IL-1α are unique in that they function as alarmins—pre-formed cytokines stored in the nucleus. Following cellular injury, alarmins are rapidly released into the extracellular space to initiate innate immune responses. IL-33 is widely regarded for its critical role in anti-parasitic immunity and allergic disease pathogenesis through activation of type 2 cytokine producing cells including group 2 innate lymphoid cells (ILC2s) and T helper 2 (Th2) cells. Ultraviolet radiation, which may worsen outcomes in patients with the allergic skin disease atopic dermatitis, was shown to induce skin fibroblast IL-33 (79). In terms of IL-1α and IL-1β, *in vitro* studies using skin, gut, and lung fibroblasts have demonstrated that fibroblasts are not only activated by IL-1 (as discussed in the previous section) but also that innate immune stimuli induce fibroblast secretion of IL-1α and IL-1β (26, 41). Functionally, IL-1 secreted by skin fibroblasts was shown to activate IL-17A producing γδT cells (26).

IL-6 is a pleiotropic cytokine essential for innate immune responses to bacteria, viruses, and parasites (48, 80). While IL-6 was one of the earliest barrier tissue fibroblast cytokines discovered (42), there is limited evidence demonstrating the importance of fibroblast-derived IL-6 in host defense beyond its initially described role in promoting T cell proliferation (42). Intriguingly, however, a recent study found that a human genetic polymorphism in the *IL6* gene has a profound effect on IL-6 production by skin fibroblasts but not by other cell types (81). This result supports previously mentioned studies that identified inter-individual differences in fibroblast activation following pattern recognition (17, 20).

G-CSF and GM-CSF are glycoprotein inflammatory mediators that support myeloid cell function (82–84). A wide variety of innate immune stimuli have been found to induce skin, gut, and lung fibroblast secretion of these mediators (32, 64, 85). Fibroblast-derived GM-CSF may be important not only for myeloid cell chemotaxis (as described above) but also for dendritic cell maturation as production of GM-CSF from an intestinal fibroblast subset was shown to be necessary for local dendritic cell education (86). Future work should investigate whether a similar mechanism applies to skin fibroblasts where location-specific fibroblast GM-CSF production has also been observed (47).

Type 1 interferons (IFN), IFNα and IFNβ, are pleiotropic antiviral cytokines produced by many cell types rapidly following PRR ligation. Barrier tissue fibroblasts are beginning to be appreciated for their ability to both produce and respond to type 1 IFN. The innate immune function of this defense system was demonstrated early on in a study using Dengue virus infected skin fibroblasts which showed that viral infection induces IFNβ that protects neighboring uninfected fibroblasts from infection (85). More recently, infection of skin fibroblasts with Zika virus was shown to induce RIG-I-dependent expression of IFN-stimulated genes and suppression of virus (9).

### Immunosuppression

Innate immune stimuli also induce fibroblast expression of immunosuppressive mediators. In the 1980s, Dayer and Elias discovered that TNFα and IL-1 activate barrier tissue fibroblasts to secrete the cyclooxygenase (COX) metabolite PGE2 (39, 87). The role of this bioactive lipid mediator in host defense is complex; generally, however, PGE2 increases susceptibility to infection and does so by inhibiting neutrophils, macrophages, and Th1 cells (88). Indeed, lung fibroblast-derived PGE2 promotes monocyte production of anti-inflammatory IL-10 (89), and intestinal fibroblast-derived PGE2 inhibits the development of inflammatory macrophages (90). More recently, a study investigating PGE2 production in fibroblasts across tissues (including intestine but not skin) found that lung fibroblasts secrete significantly more PGE2 compared to fibroblasts from to other tissues (91). Further, this report demonstrated that lung fibroblasts use PGE2 to suppress dendritic cell antigen presentation. Additional *in vitro* studies have suggested that fibroblasts can tamp down immune responses through expression of the immune checkpoint transmembrane proteins PD-L1 and PD-L2 and suppression of T cell IL-2 production and proliferation (92). TLR4 activation has been shown to further induce fibroblast PD-L1 expression and Th1 suppression (93). Notably, a multipotent and immunosuppressive subset of fibroblasts was discovered in the early 1970s and has since been referred to as mesenchymal stem/stromal cells (MSCs) (94). The immunosuppressive function of fibroblasts known as MSCs is currently being leveraged in adoptive transfer-based clinical trials for inflammatory diseases including IBD. Most of the preclinical and clinical work related to MSC immunosuppression has relied on cells derived from tissues other than skin, gut, and lung, and is thus beyond the scope of this review but has been reviewed elsewhere (95).

Collectively, these reports demonstrate that barrier tissue fibroblasts exhibit innate immune effector functions generally attributed to classical immunocytes. Immune acting fibroblasts kill bacteria and recruit and activate other cells important for defense. Further, the work highlighted above paints a picture of fibroblasts as immunological governors, equipped with the machinery to ramp up or down immune responses to best defend the host. Precisely which factors dictate whether a given fibroblast will take on more of a pro- versus anti-inflammatory state is an exciting area for future investigation. Will it be due to specific innate immune stimuli, chronic stimulation, or something else entirely? Critically, studies going forward should aim to directly compare the immune function of fibroblasts derived from different anatomical regions to better understand fibroblast heterogeneity and tissue-specificity.

## Evidence of fibroblast immune function *in vivo*

**Figure 4:**
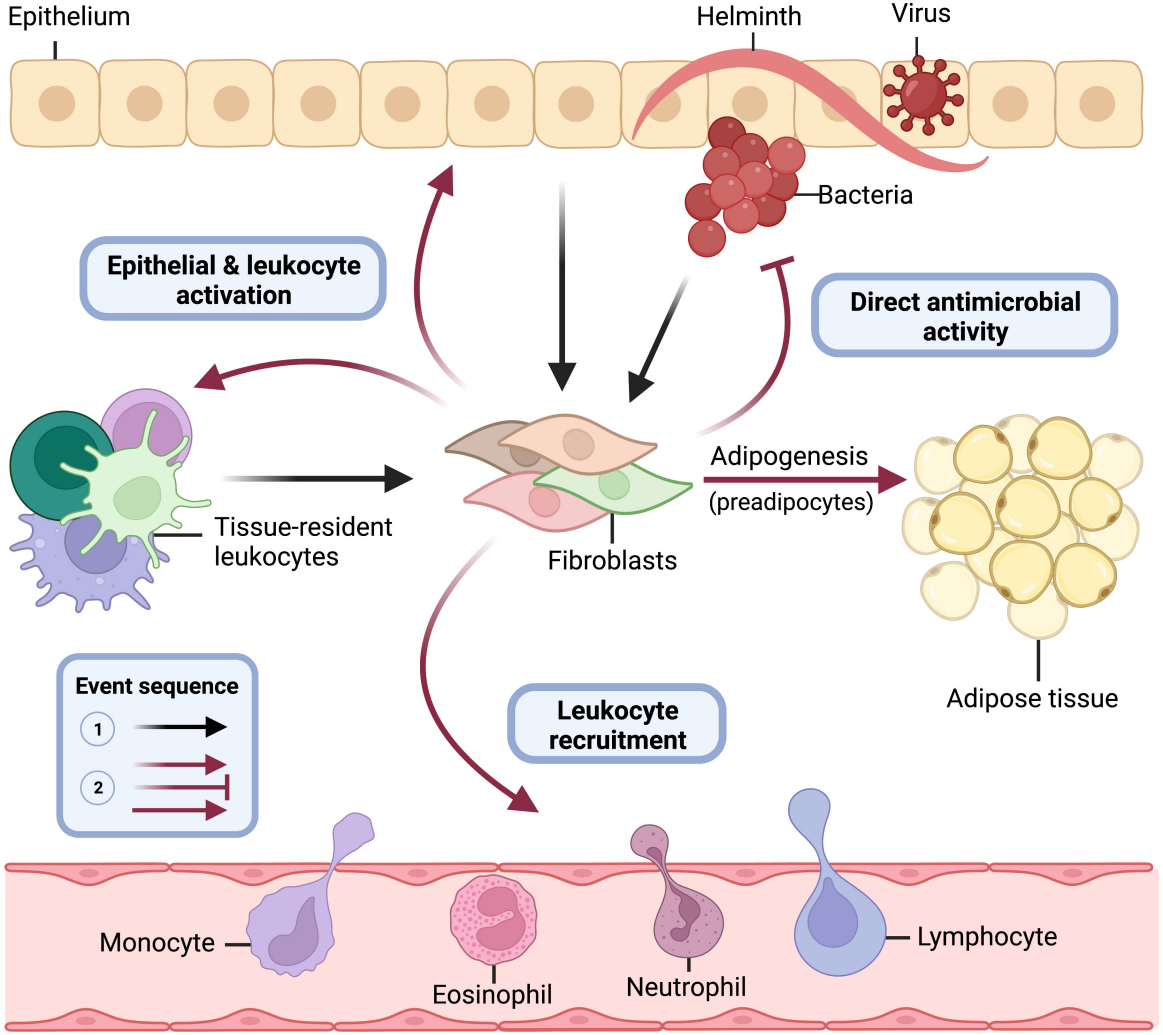
Central role of immune acting fibroblasts in innate immunity. Barrier tissue fibroblasts are tissue-resident sentinel cells, and some orchestrate innate immune responses. Following epithelial damage and breach by bacterial, viral, or parasitic pathogens, fibroblasts recognize threats directly through pattern recognition receptors (i.e., TLRs) and receive proinflammatory cytokine danger signals (i.e., TNFα) from epithelial cells and tissue-resident lymphoid and myeloid cells (black lines). Upon activation by these innate immune stimuli, committed preadipocyte fibroblasts differentiate into adipocytes (solid red line) and elicit critical immune effector functions for pathogen elimination (faded red lines) including direct antimicrobial activity, leukocyte recruitment, and activation of epithelial cells and tissue-resident leukocytes. This figure models the role fibroblasts play in innate immunity at barrier tissue sites and is not meant to suggest that each fibroblast subtype and state across tissues is known to perform all functions depicted.

Residing in the tissue and armed with sentinel immune capabilities, barrier tissue fibroblasts are positioned to be key players in innate immunity (**Figure 4**). Importantly, however, many of the immune functions described for fibroblasts could presumably drive the development of inflammatory disease if left unchecked. In this section, we will describe evidence that is now emerging from *in vivo* systems that demonstrates the importance of fibroblast immune function in the skin, gut, and lung. Much of this evidence has been gleaned from the use of fibroblast-specific conditional knockout mice (**Table 1**).

**Table 1:** Conditional knockout mice demonstrate critical role of fibroblast immune function in barrier tissue disease.

Deletion *(fl/fl)*	Targeting strategy (Cre)	Tissue	Disease model	Outcome	Reference
*ll4ra*	*Pdgfra* ^+^	Skin	Atopic dermatitis (OVA & papain)	Reduced immune fibroblast expansion	Boothby 2021
*lkbkb*	*Prrx1^+^*	Skin	Atopic dermatitis (none)	Increased Th2 inflammation	Ko 2022
*lkbkb*	*Col1a2^+^*	Skin	Atopic dermatitis (none)	Increased Th2 inflammation	Ko 2022
*lfngr1*	*Pdgfra^+^*	Skin	Vitiligo (melanoma-Treg induced)	Reduced depigmentation	Xu 2022
*lkbkb*	*Col6a1^+^*	Intestine	Colitis-associated cancer (DSS/AOM)	Reduced cancer development	Koliaraki 2015
*Tpl2*	*Col6a1^+^*	lntestine	Colitis-associated cancer (DSS/AOM)	Increased cancer development	Koliaraki 2012
*Stat3*	*Col6a1^+^*	Intestine	Colitis-associated cancer (DSS/AOM)	Reduced cancer development	Heichler 2020
*Myd88*	*Acta2^+^*	Intestine	Colitis-associated cancer (DSS/AOM)	Reduced cancer development	Yuan 2021
*Ptgs2*	*Twist2^+^*	Intestine	Colitis-associated cancer (DSS/AOM)	Reduced cancer development	Gao 2021
*Tlr4*	*Twist2^+^*	lntestine	Colitis, acute (DSS)	Increased disease severity	Gao 2021
*Ptgs2*	*Twist2^+^*	lntestine	Colitis, acute (DSS)	Increased disease severity	Gao 2021
*Mapk14*	*Twist2^+^*	Intestine	Colitis, acute (DSS)	Increased disease severity	Gao 2021
*lkkb*	*Col6a1^+^*	Intestine	Colitis, acute (DSS)	Reduced disease severity	Koliaraki 2015
*Tpl2*	*Col6a1^+^*	Intestine	Colitis, acute (DSS)	Increased disease severity	Roulis 2014
*Myd88*	*Acta2^+^*	Intestine	Colitis, acute (DSS)	Increased disease severity	Yuan 2021
*Myd88*	*Col1a2^+^*	Intestine	Colitis, acute (DSS) & homeostasis	Reduced fibroblast PD-L1 & increased IFNγ	Beswick 2014
*Myd88*	*Acta2^+^*	Intestine	Colitis, chronic (DSS)	Reduced disease severity	Zhao 2020
*Tnfsf11*	*Twist2^+^*	Intestine	Homeostasis	Reduced antigen sampling and microbial diversity	Nagashima 2017
*Tnfrsf1a*	All cells except *Col6a1^+^*	Intestine	Ileitis (TNFα over-expression)	Fibroblast TNFα signaling sufficient for pathology	Armaka 2008
*Myd88*	*Col6a1^+^*	lntestine	Spontaneous tumorigenesis (ApcMin)	Reduced cancer development	Koliaraki 2019
*Tlr4*	*Col6a1^+^*	Intestine	Spontaneous tumorigenesis (ApcMin)	Reduced cancer development	Kdiaraki 2019
*Ptgs2*	*Col6a1^+^*	Intestine	Spontaneous tumorigenesis (ApcMin)	Reduced cancer development	Roulis 2020
*Tnfrsf1a*	All cells except *Col6a1^+^*	Intestine	Viral infection (Coronavirus)	Reduced antiviral lgA production	Prados 2021
*ll15*	*Ccl19^+^*	Intestine	Viral infection (Mouse Hepatitis Virus)	Reduced ILC1s	Gil-Cruz 2016
*Myd88*	*Ccl19^+^*	Intestine	Viral infection (Mouse Hepatitis Virus)	Increased disease severity	Gil-Cruz 2016
*lgf1*	*Gli1^+^*	Lung	Bacterial infection (*S. pneumoniae*)	Reduced protection from infection	Oherle 2020
*ll17ra*	*Twist2^+^*	Lung	Bacterial vaccination *(K. pneumoniae*)	Reduced vaccine efficacy	lwanaga 2021
*ll17ra*	*Col1a2^+^*	Lung	Bacterial vaccination *(K. pneumoniae*)	Reduced vaccine efficacy	Iwanaga 2021
*ll33*	*Gli1^+^*	Lung	Helminth infection *(N. brasiliensis*)	Reduced Th2 cell development	Dahlgren 2019
*ll33*	*Ccl19^+^*	Lung	Viral infection (Adenovirus)	Reduced infiating memory CD8 T cell function	Cupovic 2021

DSS, Dextran sodium sulfate; AOM, Azaxymethane.

### Fibroblast immune function in the skin

Subsets of fibroblasts in the skin have recently been shown to be important for defense against bacterial infection and contribute to type 1 and type 2 inflammation. *S. aureus* is responsible for most skin and soft tissue infections in humans (96). In 2015, our group observed that intradermal *S. aureus* infection causes dermal *Dlk* ^+^ preadipocyte fibroblasts to differentiate into adipocytes (59). We refer to this process as “reactive adipogenesis”, and we found that fibroblasts undergoing reactive adipogenesis secrete large amounts of the antimicrobial peptide cathelicidin. Genetic or pharmacological inhibition of adipogenesis demonstrated that cathelicidin production by this process is necessary for adequate host defense against *S. aureus*. We later showed that TGFβ-mediated suppression of adipogenic fibroblast antimicrobial activity underlies the increased susceptibility to *S. aureus* infection seen in obese and elderly individuals (62, 63). *C. acnes*, another skin dwelling bacterium, is associated with acne—one of the most prevalent human skin conditions (97). Intradermal *C. acnes* infection induces perifollicular reactive adipogenesis, and experiments with human acne biopsies indicated that isotretinoin, a mainstay treatment for severe acne, likely works by amplifying fibroblast cathelicidin production (5).

There is increasing *in vivo* evidence that the immune functions of fibroblasts are important in inflammatory skin disease. Boothby and colleagues found that type 2 inflammatory events (T_reg_ depletion, helminth infection, and allergen challenge) early in life drive inflammatory crosstalk between Th2 cells and a novel eosinophilic fasciitis-associated *Pdgfra* ^+^ (a pan-fibroblast marker) fibroblast population that responds to Th2 cytokines and produces IL-33 (98). Paradoxically, Ko and colleagues discovered that mice harboring a ventral fibroblast-specific (*Prrx1* ^+^) deletion of *Ikbkb*—a key gene in the NF-κB signaling pathway that is generally proinflammatory—develop spontaneous atopic dermatitis-like Th2 inflammation (99). Fibroblasts have also been shown to play a role in Th1-skewed inflammatory disease. By performing scRNAseq on vitiligo patient biopsies and experimental vitiligo mouse models with *Ifngr1*-deficient *Pdgfra* ^+^ fibroblasts, Xu and colleagues found that fibroblasts activated by IFNγ mediate CD8 T cell recruitment underlying autoimmune depigmentation (23). Other scRNAseq studies have suggested that fibroblasts may also contribute to type 1 inflammation in cutaneous lupus erythematosus as well as type 17 inflammation during wound healing and psoriasis (100–102). Finally, it is worth reiterating that the mechanisms by which fibroblasts act as immune cells to regulate inflammatory disease pathogenesis are somewhat tissue-specific. In terms of type 2 inflammatory disease, skin fibroblasts, unlike lung and gut fibroblasts, are primed to prevent the activation of mast cells—an important cell type for the development of atopic dermatitis (103).

### Fibroblast immune function in the gut

Fibroblasts in the gut are beginning to be appreciated for their vital roles in immune development and homeostasis, host defense, and immunosuppression. During steady-state conditions, intestinal *Twist2* ^+^ (also known as *Dermo1*) fibroblasts located in the subepithelial dome of gut-associated lymphoid tissues (GALT) secrete RANKL necessary for epithelial sampling of luminal antigens, plasma cell IgA production, and maintenance of gut microbiome diversity (104, 105), and *Ccl19* ^+^ fibroblasts in the Peyer’s patches (a type of GALT) generate IL-7 and IL-15 required for homeostasis of ILC1s (106, 107).

Recent evidence suggests that immune acting fibroblasts also play important roles during infection. In response to *Salmonella* infection, pericryptal fibroblasts release IL-33 that drives epithelial cell antimicrobial activity (108). For defense against enteropathogenic viral infection, *Ccl19* ^+^ fibroblasts activated by TNFα induce plasma cell production of antiviral IgA (109). Following *Citrobacter rodentium* infection, CCL2 is upregulated, and stromal cells are the major source. In the absence of *Ccl2*, mice demonstrate reduced monocyte recruitment and pathogen clearance (12).

Because pathogens are heterogenous in terms of their susceptibility to specific immune responses, fibroblast host defense responses to one pathogen may increase the risk of infection by another. For example, when *Myd88* is deleted in *Ccl19* ^+^ fibroblasts, these cells ramp up IL-15 production and activation of antiviral ILC1s, thereby protecting mice against mouse hepatitis virus; however, increased ILC1 activation also drives post-viral dysbiosis, inflammatory disease, and sensitivity to bacterial infection (106).

Fibroblasts may also be important in IBD, which represents a collection of intestinal inflammatory conditions, including Crohn’s disease (CD) and ulcerative colitis (UC), wherein a damaged and permeable intestinal epithelium facilitates microbial translocation. Intriguingly, patients with CD often present during resection surgery with mesenteric adipose tissue projections onto inflamed intestinal tissue called “creeping fat” (110). Our group found that intestinal injury drives reactive adipogenesis and cathelicidin production and that inhibition of fat cell differentiation limits microbial translocation (111). Thus, creeping fat appears to represent the aftermath of extensive fibroblast-mediated host defense.

The innate immune role of fibroblasts in IBD extends beyond cathelicidin production. Using experimental colitis models and IBD patient samples, studies have shown that fibroblasts in the intestine produce an array of proinflammatory cytokines and chemokines (112, 113). Specifically, intestinal fibroblasts may exert direct antimicrobial activity through production of the antimicrobial lipocalin 2 and promote epithelial cell-mediated antimicrobial activity though production of IL-33. To put the brakes on protracted inflammatory responses, specific intestinal fibroblast subsets (*Twist2* ^+^ but not *Acta2* ^+^) produce PGE2 and express PD-L1 and PD-L2 (90, 92, 93, 114). Intriguingly, fibroblasts in CD express more PD-L1 than healthy control fibroblasts, while fibroblasts in UC express less PD-L1 than healthy controls (115–117).

Understanding how fibroblasts are turned on in IBD is an area of active investigation. Emerging data suggests that the mechanisms underlying fibroblast immune activation in intestinal inflammatory disease are complex and disease- and fibroblast subset-specific. For example, acute colitis is ameliorated in mice with a *Col6a1* ^+^ (a pan-fibroblast marker) fibroblast-specific deletion of *Ikbkb* (a positive regulator of proinflammatory NF-κB signaling) (118). However, other studies using mice with an *Acta2* ^+^ (basement membrane adjacent) fibroblast-specific deletion of *Myd88* (also a positive regulator of NF-κB signaling) found that acute colitis was exacerbated but that chronic colitis was ameliorated (119, 120).

In terms of upstream innate immune stimuli, it has been observed that mice with the TNFα receptor 1 (TNFR1) restricted to *Col6a1* ^+^ fibroblasts develop pathology in a TNFα-induced model of Crohn’s disease (121). This finding further suggests that biologic TNFα inhibitors, a mainstay treatment in the IBD pharmacological armamentarium, may work in part by inhibiting proinflammatory fibroblast activity. Unfortunately, 30% of patients do not respond to anti-TNFα treatment. In these patients, OSM and IL-1 have been shown to be dominant drivers of intestinal fibroblast production of proinflammatory cytokines and chemokines (46, 50). These findings, among others, have generated large interest in OSM and IL-1 inhibitors as potential therapeutic strategies for IBD.

Emerging evidence indicates that immune acting fibroblasts also participate in intestinal tumorigenesis. Colon cancer may arise spontaneously or due to unresolved inflammation. In spontaneous models of tumorigenesis, mice with *Col6a1* ^+^ fibroblast-specific deletions of *Tlr4*, *Myd88*, or *Ptgs2* (encodes an enzyme important for PGE2 production) exhibit reduced cancer development (10, 118, 122). In colitis-associated cancer (CAC) models, mice with *Col6a1* ^+^ fibroblast-specific deletion of *Stat3 or Ikbkb* (but not *Tlr4* or *Myd88*) demonstrate reduced cancer development (10, 118, 123, 124). Interestingly, when *Myd88* is deleted specifically in *Acta2* ^+^ fibroblasts, mice exhibit reduced CAC development (120). *Twist2* ^+^ fibroblasts have also been targeted in CAC models. When *Ptgs2* is deleted in this fibroblast subset, mice demonstrate reduced CAC (90). This finding, coupled with the results from the *Col6a1* ^+^ fibroblast-specific *Ptgs2* knockout mouse mentioned above, paints a picture of fibroblast-derived PGE2 as a powerful tumor promoter. Another fibroblast-derived immune mediator that plays a role in cancer development is the IL-6 family member IL-11. A recent study showed that IL-11 is upregulated in CAC and that a subpopulation of fibroblasts lacking αSMA is the major source (125). Further, in the absence of IL-11 (or its receptor) mice were protected from cancer development. Collectively, these reports suggest that fibroblast immunosuppressive activity generates an environment permissive for cancer growth, though the precise mechanisms underlying fibroblast immune activity in cancer are, as in IBD, complex.

While most of the work regarding gut fibroblast immune function has focused on the intestines, there is a growing body of work surrounding the role of immune acting fibroblasts in the esophageal and oral mucosae. In the allergic disease eosinophilic esophagitis, the overexpressed TNF superfamily member LIGHT has been shown to drive proinflammatory fibroblast responses (126–128), and a recent scRNAseq study of the oral mucosa suggested that fibroblasts may drive neutrophil infiltration in health and periodontal disease (129).

Taken together, this large body of work on gut fibroblasts demonstrates that specific populations are important for development of the immune system, defense against pathogens, and timely immunosuppression; however, these cells must be tightly regulated to not inadvertently contribute to pathogenesis.

### Fibroblast immune function in the lung

Fibroblasts in the lung are emerging as important cells in pulmonary host defense against bacteria, viruses, and parasites. Using mice with IL-17R genes specifically deleted in *Col1a2* ^+^ (a pan-fibroblast marker) or *Twist2* ^+^ fibroblasts, IL-17 signaling in fibroblasts was shown to be important for vaccine efficacy against *K. pneumoniae* (130). Another study found that adoptive transfer of Sca-1^+^ fibroblasts in a mouse model of *K. pneumoniae*-induced pneumonia suppresses bacterial growth and improves survival (66). Lung fibroblasts also function upstream of Th17 responses. Alveolar *Gli1* ^+^ fibroblasts produce IGF1 critical for the development of ILC3s and protection against *Streptococcus pneumoniae* (131). For defense against metazoan parasites and viruses, perivascular *Gli1* ^+^ fibroblast-derived IL-33 is required for the development of helminth-induced Th2 cells (132), and perivascular *Ccl19* ^+^ fibroblasts (in the lung but not lymphoid tissue) produce IL-33 necessary for adenovirus-induced inflating memory CD8 T cell development and function (133). Notably, some lung pathogens have evolved mechanisms to subvert fibroblast immune responses for survival. For example, a recent study showed that *Mycobacterium tuberculosis* invades Sca-1^+^CD73^+^ lung fibroblasts and drives up fibroblast PGE2 production, thus protecting itself from immune- and drug-mediated killing (134).

Like gut fibroblasts, specific lung fibroblast subsets have been shown to play roles in cancer and immune suppression. *Ccl19* ^+^ perivascular lung fibroblasts were found to be essential for leukocyte chemotaxis and protection against cancer (135). Other studies have discovered detrimental roles for fibroblasts in cancer. Specifically, a Sca-1^+^CD44^+^ fibroblast subset recruit and activate neutrophils using C3a, thereby creating a niche for metastasis (70). *Pdgfra* ^+^ fibroblast *Ptgs2* also promotes metastasis (91). In terms of immunosuppression, it was recently observed that mice with a *Twist2* ^+^ fibroblast-specific deletion of *Ryk* (co-receptor for Wnt) develop spontaneous lung inflammation (136).

Collectively, these studies illustrate that specific populations of lung fibroblasts can act as immune cells and are important for the development of several branches of the immune system, defense against a diverse array of clinically relevant pathogens, and regulation of inflammation and tumorigenesis.

## Discussion

Fibroblasts were once thought to represent a homogenous group of structural cells but are now appreciated as a diverse and multifunctional class of cells. Immune functions have been clearly detected in subsets of fibroblasts, and these activities appear to be important for host defense. Fibroblasts located in the subepithelial layers of the skin, gut, and lung likely serve as sentinel immune cells that respond to pathogens and elicit innate immune effector functions including direct antimicrobial activity, leukocyte recruitment, and cytokine production. Importantly, the precise outcome of immune acting fibroblasts varies depending on several factors including the inflammatory insult, the fibroblast subset, and its environment.

Historically, most studies revealing fibroblast function have done so using populations of cells expanded *in vitro* without a complete understanding of the diversity and multifunctionality of cells called ‘fibroblasts’. Future work should build on recent single-cell analysis efforts to characterize markers that identify subsets of fibroblasts and define their lineages, activation states, and spatial locations. Such studies will facilitate discussion of immune acting fibroblasts and guide future research. Because some innate immune functions are conserved between fibroblasts from different barrier tissues, future work should address whether findings related to fibroblasts in one tissue apply to another. For example, do fibroblasts in the lung undergo reactive adipogenesis like fibroblasts in the skin and gut? And do skin and gut fibroblasts exhibit features of trained immunity like fibroblasts in the lung? Moreover, ongoing efforts should elucidate whether fibroblast immune functions that have been identified in barrier tissues also apply to non-barrier tissue diseases, such as rheumatoid and psoriatic arthritis. For example, does lung fibroblast trained immunity contribute to chronic inflammatory disease exacerbation and recurrence as synovial fibroblast trained immunity does in rheumatoid arthritis (137)? Finally, while the list of mediators discovered to activate fibroblasts grows long, our understanding of inhibitory molecules is severely lacking. Overall, with a better understanding of fibroblast biology, immune acting fibroblasts may ultimately be specifically targeted or used in adoptive transfer cell-based therapies for treatment of infection, inflammatory disease, and cancer.

## Data availability statement

The original contributions presented in the study are included in the article/supplementary material. Further inquiries can be directed to the corresponding author.

## Author contributions

KC and RG wrote and edited the manuscript. All authors contributed to the article and approved the submitted version.
